# The Role of Genetic Analysis in Demystifying the Diagnosis in a Middle-Aged Male Presenting With Proximal Muscle Weakness and Sclerotic-Lytic Skeletal Lesions

**DOI:** 10.7759/cureus.50924

**Published:** 2023-12-21

**Authors:** Soham Mukherjee, Karthik V Mahesh, Sanjay K Bhadada, Debajyoti Chatterjee, Rajender Kumar

**Affiliations:** 1 Endocrinology, Postgraduate Institute of Medical Education and Research, Chandigarh, IND; 2 Neurology, Postgraduate Institute of Medical Education and Research, Chandigarh, IND; 3 Pathology, Postgraduate Institute of Medical Education and Research, Chandigarh, IND; 4 Nuclear Medicine, Postgraduate Institute of Medical Education and Research, Chandigarh, IND

**Keywords:** sclerotic-lytic lesions, vcp gene, ibmpfd, inclusion body myopathy, paget’s disease of bone

## Abstract

Paget's disease of bone (PDB) usually presents with bone pain and deformities. Herein, we describe a case of PDB who presented with gradually progressive quadriparesis. A man in his forties presented with gradually progressive proximal muscle weakness involving all four limbs. The patient had an elevated serum alkaline phosphatase level and osteosclerosis at various skeletal sites in a radiological skeletal survey. 18F-fluorodeoxyglucose (FDG) PET-CT showed FDG-avid sclerotic-lytic lesions at multiple skeletal sites. Histopathology evaluation of bone and muscle biopsy specimens revealed PDB and inclusion body myopathy (IBM) with neurogenic atrophy, respectively. A diagnosis of IBM associated with PDB without frontotemporal dementia (IBMPFD) was suspected and confirmed by exome sequencing, which revealed a heterozygous mutation in the *VCP* gene. The bone disease responded to zoledronate administration. A high index of suspicion for IBMPFD should be kept in mind in any patient with PDB presenting with proximal muscle weakness.

## Introduction

Gene panels are regularly employed by specialized neuromuscular clinics to identify rare causes of muscular dystrophies, especially if the clinical patterns are not typical for the most common types in that population subgroup [1]. This increased use has been attributed to wider availability, acceptability, and acceptance as it is less invasive than a muscle biopsy [1].

Inclusion body myopathy (IBM) is a myopathy predominantly affecting elderly males in the fifth to sixth decade. It is characterized by predominant asymmetrical weakness of long flexors of the forearm and knee extensors. Though long considered an inflammatory myopathy, recent evidence shows it could be a part of a neurodegenerative disorder given its elderly onset, male predominance, and poor response to immunotherapy [2]. When associated with valosin-containing protein (VCP), it consists of proximal myopathy involving both lower and upper extremities [2]. Patients even have lordotic stances and later can have distal muscle, cardiac, and respiratory muscle involvement. This pattern of weakness may mimic late-onset limb-girdle muscular dystrophy or fascioscapular muscular dystrophy, leading to a delay in diagnosis.

Paget’s disease of bone (PDB) is a metabolic bone disease and is characterized by focal areas of increased osteoclastic bone resorption coupled with increased but disorganized osteoblastic bone formation [3]. The most common clinical manifestations are bone pain and deformities. Rarely, these patients may also present with muscular weakness.

Spine involvement in PDB usually results in bone pain, and more severe cases may lead to compressive myelopathy or radiculopathy leading to muscular weakness [4]. Sometimes, shunting of blood from spinal arteries to highly vascular bones may lead to paraparesis [4,5]. Very rarely, PDB can manifest as a component of an uncommon autosomal dominant progressive disorder known as IBM with PDB with or without frontotemporal dementia (FTD) (IBMPFD), where the patient may present with progressive quadriparesis [6]. In 2004, missense mutations in the valosin-containing protein (VCP) gene were reported to be causally linked with IBMPFD [7]. VCP is ubiquitously expressed and involved in proteasomal and autophagic pathways mediated protein degradation [8]. Since then, about 70 IBMPFD families have been described in the literature, along with at least 35 different mutations in the VCP [9]. Herein, we describe a case of PDB who presented with gradually progressive quadriparesis and was eventually found to have IBMPFD.

## Case presentation

A 48-year-old male patient presented with weakness in both upper and lower limbs for the past four years and low backache for the last two years. Initially, he noticed difficulty in climbing upstairs, getting up from a sitting position, walking, combing his hair, and raising his arms above his shoulders. He did not complain of slippage of footwear or difficulty in buttoning or unbuttoning. However, there were no sensory symptoms. Bowel and bladder function was normal. There was no past history of fracture, bony swelling, or deformity. He was diagnosed with Type 2 diabetes three months ago, detected incidentally during routine blood investigation, and initiated on the oral anti-diabetic agent metformin. There was no similar history in the family. On examination, he had proximal muscle weakness involving bilateral hip extensors, knee extensors, and flexors. In the upper limbs, there was bilateral scapular winging, deltoid, biceps, abductor pollicis brevis, and dorsal interossei wasting. His reflexes, including bilateral supinators, biceps, triceps, knee, and ankle, were sluggish and present only with Jendrassik's (1+) (normal 2+) in the upper limbs and were absent in the lower limbs with bilateral flexor plantar reflexes, suggestive of a combined myopathic and neurogenic process. There was no clinical evidence of dementia.

Investigations

**Figure 1:**
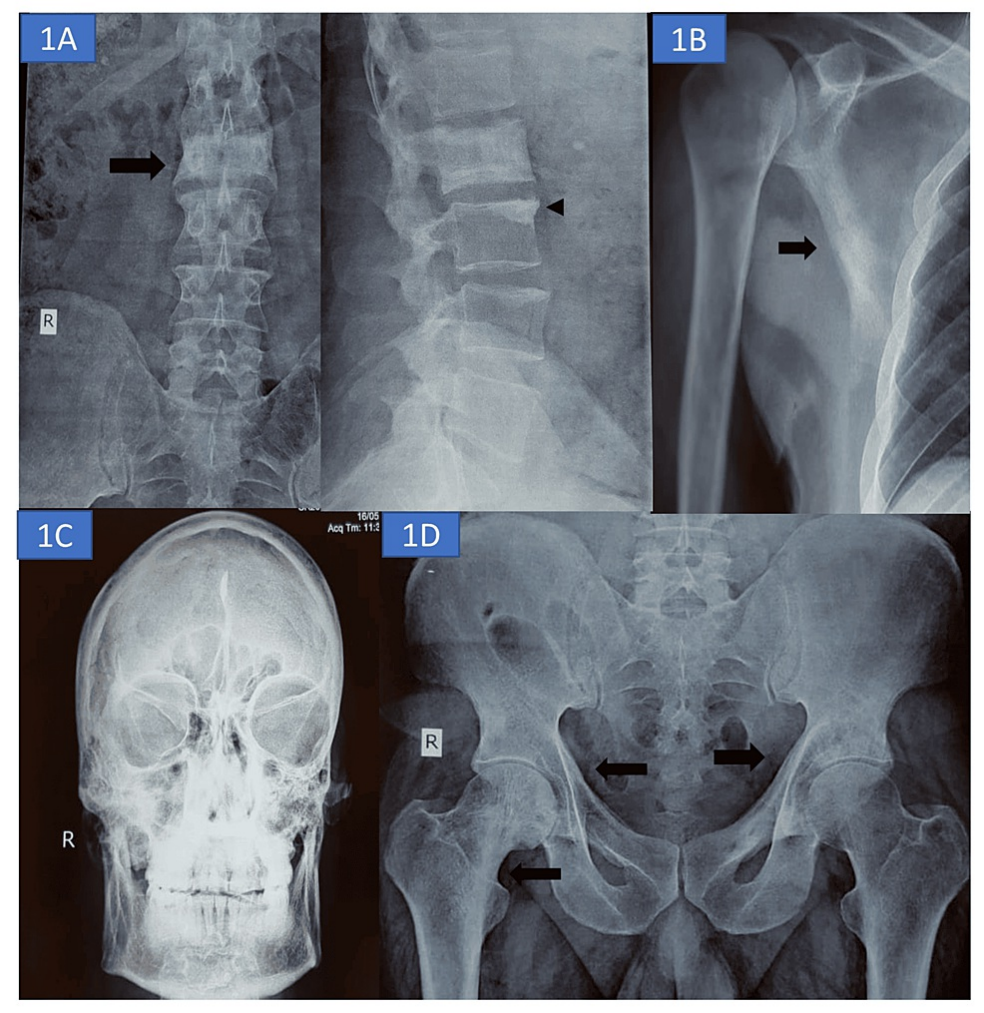
Skeletal radiograph (A) X-ray of the lumbar spine AP and lateral view shows diffuse sclerosis of the L2 vertebral body (ivory vertebra) with coarsened trabeculae (arrow). Degenerative sclerosis of the L3 upper end plate is also seen (arrowhead). (B) Cortical thickening of the lateral scapular margin is seen in the right shoulder. (C) X-ray of the skull AP view shows diffuse widening, thickening, and sclerosis of the diploic spaces of the skull bones. (D) X-ray of the pelvis AP view shows thickened bilateral iliopectineal line and coarsened trabeculae of the inferomedial right femoral neck (black arrows).

**Figure 2:**
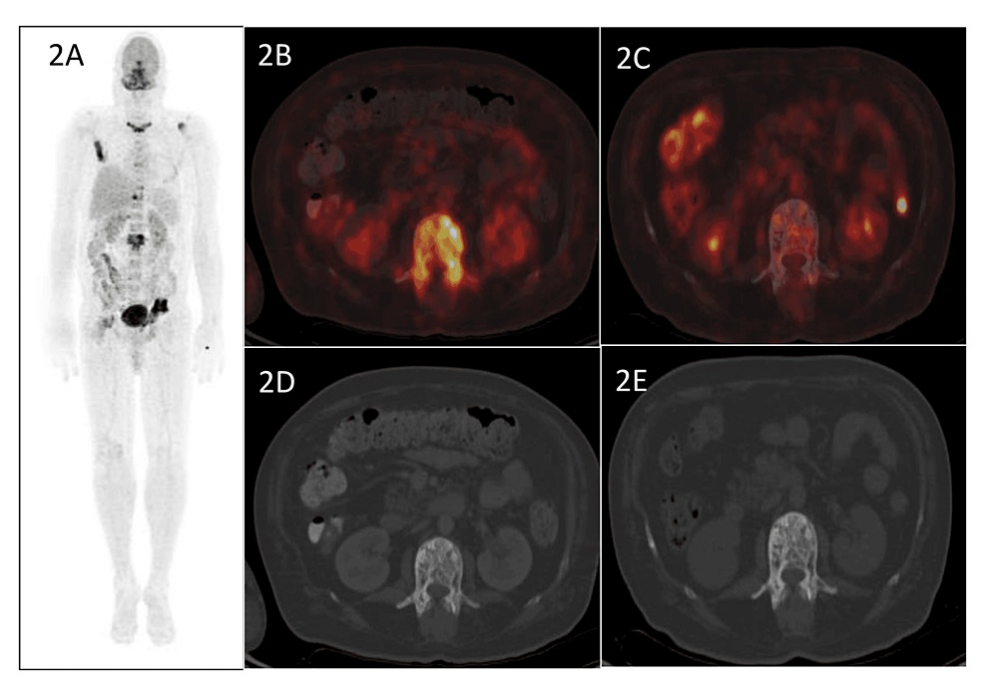
18F-FDG PET/CT (A) Fluorodeoxyglucose (FDG) PET/CT shows multiple abnormal foci of tracer uptake in the regions of the skull, sternum, right scapula, a few vertebrae, region of left shoulder, left hemipelvis, and right femur. (B and D) Trans-axial fused PET/CT and CT images localize the tracer uptake to lytic sclerotic changes in the L2 vertebra (SUV max – 11.1). (C and E) A regional FDG PET/CT done following zoledronate infusion shows the resolution of FDG uptake in the L2 vertebra on the PET/CT images with no significant anatomical change on the CT images.

Blood investigations have been detailed in Table 1. The calcium profile was normal, except for an elevated total alkaline phosphatase (ALP) level. The bone turnover markers carboxy-terminal collagen crosslinks (CTX) and procollagen 1 N-terminal propeptide (P1NP) were also raised. Given the low backache and raised ALP level, a radiological skeletal survey was conducted, which revealed osteosclerosis involving the skull, scapulae, lumbar vertebrae, pelvic bones, and both femurs (Figure 1A-D). The nerve conduction study (NCS) was suggestive of sensorimotor axonal polyneuropathy. Electromyography (EMG) was suggestive of a myogenic pattern, and NCS-EMG was corroborative of the clinical findings. Serum protein electrophoresis did not reveal an M spike, and urine protein electrophoresis was also not suggestive of monoclonal gammopathy. Other tumor markers, including serum prostate-specific antigen, alpha-fetoprotein, carcinoembryonic antigen, and beta HCG level, were also within normal limits. MRI spine did not reveal any evidence of cord or nerve compression but showed lytic lesions in the vertebrae. An 18F-fluorodeoxyglucose (FDG) PET-CT was done, which showed FDG-avid, predominantly sclerotic, multiple sclerotic-lytic lesions at the body and greater wing of the sphenoid, petrous part of the temporal bone, occipital bones, both scapulae, left 5th rib, right 12th rib, body and posterior elements of a few dorso-lumbar vertebrae, pelvic bones on both sides, and right femur (Figure 2A, B, and D).

**Table 1:** Hematological and biochemical profile

Parameters	Value	Reference range
Hemoglobin (g/dL)	15	12–17
Total leucocyte count (cells/mm^3^)	6600	4000–11000
Platelet count (cells/mm^3^)	198,000	150,000–450,000
Creatinine (mg/dL)	0.5	0.4–1.1
Calcium (mg/dL)	9.9	8.6–10.2
Phosphorus (mg/dL)	3.4	2.5–4.5
Albumin (g/dL)	4.7	3.5–5.5
Alkaline phosphatase, ALP (U/L)	878	40–129
25(OH) vitamin D (ng/mL)	32	30–100
Intact parathyroid hormone (pg/mL)	30	15–65
Glycosylated hemoglobin (%)	5.8	≤5.6
Carboxy-terminal collagen crosslinks, CTX (pg/mL),	988	300±142
Procollagen I N-terminal propeptide, P1NP (ng/mL)	491	48.2±18.1
Creatinine kinase (U/L)	118	46–171

**Figure 3:**
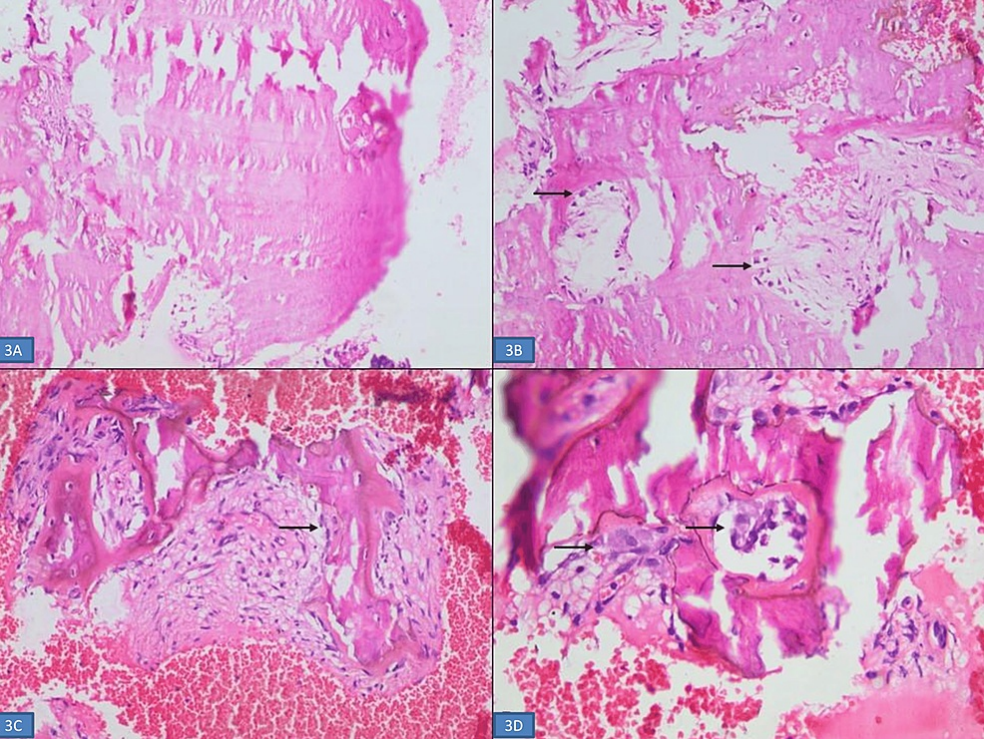
Bone biopsy (A) Areas of thick lamellar bone (hematoxylin and eosin, x100). (B) Areas showing lamellar bone with osteoblastic activity, indicated by the black arrow (hematoxylin and eosin, x100). (C) New bone formation with osteoblastic activity, indicated by the black arrow (hematoxylin and eosin, x200). (D) Areas of bone resorption with osteoclastic activity, indicated by the black arrow (hematoxylin and eosin, x200).

**Figure 4:**
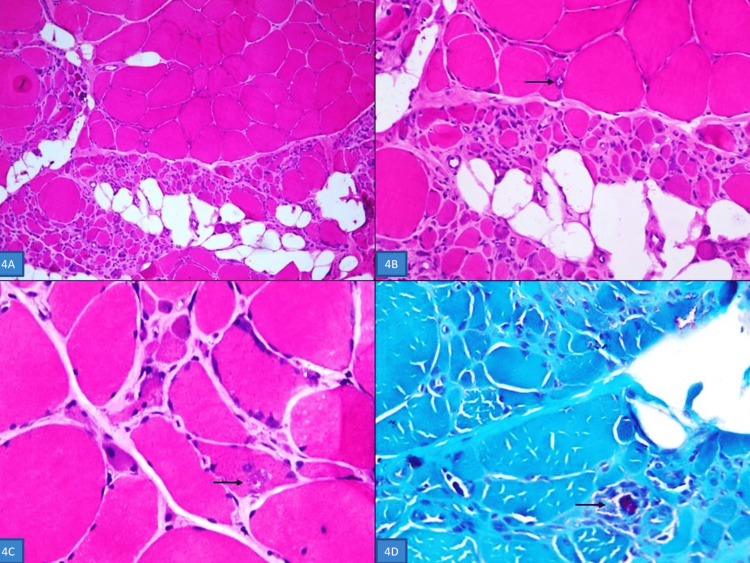
Muscle biopsy (A) Snap-frozen muscle biopsy shows panfascicular atrophy with fat infiltration in the lower half, indicating neurogenic atrophy (hematoxylin and eosin, x40). (B) Higher magnification shows an inclusion with a rimmed vacuole (black arrow) (hematoxylin and eosin, x100). (C) Another fiber showing an inclusion with a rimmed vacuole (black arrow) (hematoxylin and eosin, x200). (D) The inclusions are highlighted by modified Gomori's trichrome stain (x200).

Histopathology evaluation of PET-guided bone biopsy specimen from the left iliac bone revealed features suggestive of PDB (Figure 3A-D). Snap-frozen muscle biopsy was suggestive of Inclusion Body Myopathy (IBM) with neurogenic atrophy (Figure 4A-D). A diagnosis of IBM associated with PDB without frontotemporal dementia (IBMPFD) was suspected and confirmed by clinical exome sequencing (with the Illumina sequencing platform), which revealed a heterozygous mutation in the VCP gene [c.463C>T (p.Arg155Cys)].

Treatment, outcome, and follow-up

Zoledronate (5 mg) was administered intravenously, which resulted in marked clinical and biochemical improvement in the PDB component of the syndrome, with normalization of ALP level (112 IU/L) and reduction in tracer avidity in repeat 18F-FDG PET-CT three months after zoledronate infusion (Figure 2C and E). Physiotherapy for myopathy was initiated as there is no role for steroids or immunotherapy in these cases. The patient is doing well at two years of follow-up with persistently normal serum ALP level.

## Discussion

IBMPFD is an extremely rare, multi-system disorder with autosomal dominant inheritance, first described in 1982 [10]. As the nomenclature implies, the condition is characterized by three main clinical phenotypes with variable expressivity including IBM, PDB, and FTD [11, 12]. Other less common associations include amyotrophic lateral sclerosis (ALS), parkinsonism, neuropathy, spastic paraplegia, and cardiomyopathy. Some prefer to use the term multisystem proteinopathy (MSP) over IBMPFD due to its diverse and varying manifestations [11]. The mutation associated with the VCP gene is most commonly responsible for IBMPFD. However, other genes including hnRNPA2B1, hnRNPA1, SQSTM1, MATR3, TIA1, OPTN, ANXA11, and PFN1 may also result in similar clinical manifestations [2]. About 70 IBMPFD families have been described in the literature, along with at least 35 different mutations in the VCP gene [9]. The VCP-associated IBMPFD is an autosomal dominant, highly penetrant disorder with variable expressivity [9]. If a clinically unaffected individual has a pathogenic or likely pathogenic mutation in the VCP gene, there is almost a 90% chance that the individual will likely develop features of the disorder by 45 years of age [2].

PDB has been observed in approximately half of the patients with IBMPFD [11]. Usually, the age of onset is between 30-40 years [13]. PDB is caused by increased osteoclastic activity and increased bone turnover, leading to bone deformities and fractures. Often there is focal involvement, with the most commonly involved bone sites being the vertebrae, skull, and pelvis [13]. The diagnosis of PDB in our case was suspected based on the biochemical profile, with an elevation of serum ALP, CTX, and P1NP levels suggestive of increased bone turnover [14]. The diagnosis was further supported by FDG PET findings and confirmed by histopathology. Oral or intravenous bisphosphonates (e.g., zoledronic acid) are effective in this condition, as these agents are potent inhibitors of osteoclastic activity [15]. Our patient also responded to intravenous zoledronic acid with normalization of ALP level and marked reduction of tracer avidity in repeat 18F-FDG PET-CT image. Denosumab, another anti-resorptive agent, was effective in reducing bone pain and ALP levels in patients with PDB [16]. However, its widespread use in PDB requires more studies.

Inclusion body myopathy is the most common manifestation and has been reported in 80-90% of IBMPFD cases [13]. The usual age of onset is the third to fourth decade. There is progressive involvement of the proximal muscles, including the pelvic and shoulder girdle muscles. Gradual progression of the disease may result in respiratory and cardiac muscle involvement, and the patient may die of respiratory failure or cardiomyopathy. Serum creatinine kinase concentration is usually normal to mildly elevated, as in our case [13]. Similar to the index patient, electromyography usually shows myopathic changes. Histopathology of the involved muscle is usually diagnostic and classically shows rimmed vacuoles, and sarcoplasmic and myonuclear inclusion bodies [7]. As far as treatment is concerned, no disease-modifying therapy is available for IBM to date [2].

The third component of IBMPFD is frontotemporal dementia, which affects approximately 30% of patients with VCP-associated IBMPFD and usually presents as the behavioral variant frontotemporal dementia subtype [2]. The index case did not have any clinical evidence of frontotemporal dementia.

VCP is a member of the ATPases associated with a variety of cellular activities (AAA) protein superfamily. VCP is involved in multiple ubiquitin-dependent intracellular processes [9]. Loss of function of VCP results in cell cycle arrest, ubiquitinated protein accumulation, and intracellular vacuole formation. The derangement observed in the ubiquitination pathway mostly explains the pathological alteration seen in IBMPFD [2]. Apart from involvement in protein degradation through the ubiquitin/proteasome system, VCP also participates in various cellular processes and functions, which include surveillance of misfolded proteins, DNA repair, post-mitotic organelle reassembly, cellular apoptosis, and cell cycle regulation [13]. VCP plays a crucial role in the degradation of IκBα, a repressor of NFκB (NFκB). Hence, in VCP mutation, NFκB is upregulated, which in turn stimulates osteoclastic activity, an important pathogenic mechanism of PDB [13, 17]. NFκB pathway activation also has an important role in skeletal muscle atrophy [13].

## Conclusions

To conclude, this case stresses the importance of maintaining a high index of suspicion for IBMPFD in a patient with PDB who has evidence of myopathy, after ruling out more common causes of muscle weakness such as myeloradiculopathy, late-onset myopathies, metabolic myopathies, and inflammatory myositis.

## References

[1] Thuriot F, Gravel E, Buote C (2020). Molecular diagnosis of muscular diseases in outpatient clinics: a Canadian perspective. Neurol Genet.

[2] Korb M, Peck A, Alfano LN (2022). Development of a standard of care for patients with valosin-containing protein associated multisystem proteinopathy. Orphanet J Rare Dis.

[3] Bhadada S, Bhansali A, Unnikrishnan AG, Khadgawat R, Singh SK, Mithal A, Saikia UN (2006). Does Paget's disease exist in India?: a series of 21 patients. J Assoc Physicians India.

[4] Singer FR (2016). Endocrinology: adult and pediatric. Paget's Disease of Bone, 7th Edition.

[5] Bhadada S, Bhansali A, Singh R, Saikia UN, Khandelwal N, Sreenivasulu P (2006). An unusual endocrine cause of recurrent paraparesis: Paget disease. Endocrinologist.

[6] Kovach MJ, Waggoner B, Leal SM (2001). Clinical delineation and localization to chromosome 9p13.3-p12 of a unique dominant disorder in four families: hereditary inclusion body myopathy, Paget disease of bone, and frontotemporal dementia. Mol Genet Metab.

[7] Watts GD, Wymer J, Kovach MJ (2004). Inclusion body myopathy associated with Paget disease of bone and frontotemporal dementia is caused by mutant valosin-containing protein. Nat Genet.

[8] Meyer H, Weihl CC (2014). The VCP/p97 system at a glance: connecting cellular function to disease pathogenesis. J Cell Sci.

[9] Shinjo SK, Oba-Shinjo SM, Lerario AM, Marie SK (2018). A Brazilian family with inclusion body myopathy associated with Paget's disease of bone and frontotemporal dementia linked to the VCP pGly97Glu mutation. Clin Rheumatol.

[10] Tucker WS Jr, Hubbard WH, Stryker TD, Morgan SW, Evans OB, Freemon FR, Theil GB (1982). A new familial disorder of combined lower motor neuron degeneration and skeletal disorganization. Trans Assoc Am Physicians.

[11] Gu JM, Ke YH, Yue H (2013). A novel VCP mutation as the cause of atypical IBMPFD in a Chinese family. Bone.

[12] Kimonis VE, Kovach MJ, Waggoner B (2000). Clinical and molecular studies in a unique family with autosomal dominant limb-girdle muscular dystrophy and Paget disease of bone. Genet Med.

[13] Nalbandian A, Donkervoort S, Dec E (2011). The multiple faces of valosin-containing protein-associated diseases: inclusion body myopathy with Paget's disease of bone, frontotemporal dementia, and amyotrophic lateral sclerosis. J Mol Neurosci.

[14] Pal R, Bhadada SK (2022). Comment on “rebound hypercalcemia post-denosumab cessation in metastatic breast cancer”: Waxing-Waning serum calcium following denosumab use in a patient with polyostotic Paget’s disease of bone. Osteoporos Int.

[15] Reid IR, Miller P, Lyles K (2005). Comparison of a single infusion of zoledronic acid with risedronate for Paget's disease. N Engl J Med.

[16] Ralston SH, Corral-Gudino L, Cooper C (2019). Diagnosis and management of Paget’s disease of bone in adults: a clinical guideline. J Bone Miner Res.

[17] Daroszewska A, Ralston SH (2006). Mechanisms of disease: genetics of Paget's disease of bone and related disorders. Nat Clin Pract Rheumatol.

